# Mechanical modelling quantifies the functional importance of outer tissue layers during root elongation and bending

**DOI:** 10.1111/nph.12764

**Published:** 2014-03-18

**Authors:** Rosemary J Dyson, Gema Vizcay-Barrena, Leah R Band, Anwesha N Fernandes, Andrew P French, John A Fozard, T Charlie Hodgman, Kim Kenobi, Tony P Pridmore, Michael Stout, Darren M Wells, Michael H Wilson, Malcolm J Bennett, Oliver E Jensen

**Affiliations:** 1 School of Mathematics, University of BirminghamEdgbaston, Birmingham, B15 2TT, UK; 2 Centre for Ultrastructural Imaging, King's College LondonLondon, SE1 1UL, UK; 3 Centre for Plant Integrative Biology, University of NottinghamSutton Bonington, LE12 5RD, UK; 4 School of Physics & Astronomy, University of Nottingham, University ParkNottingham, NG7 2RD, UK; 5 School of Computer Science, University of NottinghamJubilee Campus, Nottingham, NG8 1BB, UK; 6 School of Biosciences, University of NottinghamSutton Bonington, LE12 5RD, UK; 7 School of Mathematics, University of ManchesterOxford Road, Manchester, M13 9PL, UK

**Keywords:** *Arabidopsis thaliana*, elongation, extensibility, gravitropism, mechanical modelling, multiscale

## Abstract

- Root elongation and bending require the coordinated expansion of multiple cells of different types. These processes are regulated by the action of hormones that can target distinct cell layers. We use a mathematical model to characterise the influence of the biomechanical properties of individual cell walls on the properties of the whole tissue.
- Taking a simple constitutive model at the cell scale which characterises cell walls via yield and extensibility parameters, we derive the analogous tissue-level model to describe elongation and bending. To accurately parameterise the model, we take detailed measurements of cell turgor, cell geometries and wall thicknesses.
- The model demonstrates how cell properties and shapes contribute to tissue-level extensibility and yield. Exploiting the highly organised structure of the elongation zone (EZ) of the *Arabidopsis* root, we quantify the contributions of different cell layers, using the measured parameters. We show how distributions of material and geometric properties across the root cross-section contribute to the generation of curvature, and relate the angle of a gravitropic bend to the magnitude and duration of asymmetric wall softening.
- We quantify the geometric factors which lead to the predominant contribution of the outer cell files in driving root elongation and bending.

## Introduction

The growth of elongated plant organs is typically controlled by competition between turgor pressure and targeted wall softening, allowing cells and tissues to expand in a highly regulated manner. Studies of individual cells have demonstrated how cellulose microfibrils embedded in the cell wall can promote axial elongation with minimal radial expansion, at a rate determined by the properties of the wall's pectin matrix and hemicellulose crosslinks (Cosgrove, 2000, 2005; Baskin, 2005; Boyer, 2009). In a tissue, cells adhere strongly to their neighbours, even though the mechanical properties of neighbouring cell types may differ. Elongation of an organ such as the primary root of *Arabidopsis* is therefore determined by the integrated effect of multiple cells, and will be mediated by geometric as well as biomechanical factors.

As cells traverse the elongation zone (EZ) of the *Arabidopsis* root, their growth rates change: measurements show a dramatic increase in the cell's relative elongation rate (RER) on entering the EZ; this RER is then maintained at a high level before reducing to zero as cells progress to the mature zone (van der Weele *et al.* 2003; Basu *et al.* 2007; Chavarría-Krauser *et al.* 2008). Because the root's overall elongation rate depends on the rate at which mature cells are produced and their length, the duration and growth of cells within the EZ critically affects root growth. Many genetic mutants with reduced root length have reduced cell growth within the EZ (Benfey *et al.* 1993; Band *et al.* 2012b; Wen *et al.* 2013). The regulation of growth by phytohormones is of particular significance. For example, it is recognised that different hormones target different cell layers (Swarup *et al.* 2005; Úbeda-Tomás *et al.*, 2008; Hacham *et al.* 2011), with auxin and brassinosteroid targeting the epidermis and gibberellin targeting the endodermis. This raises the question of how signals acting on different cell layers together regulate the shape of the growing root, and why particular hormones come to have a dominant influence on specific cell layers (Úbeda-Tomás *et al.*, 2012).

In building systems-level descriptions of plant organs, it is necessary to integrate the action of multiple individual components acting across disparate time and length scales (Band *et al.* 2012a). In modelling growth of the *Arabidopsis* root, a number of these components have recently been put into place. At the level of an individual cell wall, chemo-mechanical models have addressed the turnover of pectin (Rojas *et al.* 2011) and of hemicellulose cross-links that bind to cellulose microfibrils (Dyson *et al.* 2012), showing in the latter case how a stretch-dependent breakage rate can explain yielding behaviour of the wall. At the level of a single cell, a model describing the reorientation of microfibrils as a cell elongates has revealed a potential biomechanical mechanism for the suppression of cell elongation as cells leave the EZ (Dyson & Jensen, 2010). These studies demonstrate how variants of the Lockhart equation (Lockhart, 1965; Ortega, 1985) (in which cell-wall material is characterised by yield and extensibility parameters) provide a practical description of plant materials at different scales. These descriptions have been integrated into a two-dimensional representation of a multicellular plant root (Fozard *et al.* 2013), illustrating how differential expansion generates bending and microfibril reorientation inhibits growth. The value of this approach is that simulations can capture detailed biomechanical properties of cell walls and a realistic representation of multicellular tissue geometry, while being coupled to descriptions of hormone transport and signalling pathways between and within individual cells.

In the development of simulations of this kind, techniques from multiscale modelling enable us to connect representations of a system across different spatial scales, providing mechanistic insights in addition to significant computational advantages. Here we pursue such an approach, seeking to understand how the mechanical properties of individual cells over the cross-section of an elongating organ such as a root contribute to the properties of the tissue as a whole, particularly in driving morphometric changes such as gravitropic bending. While a Lockhart-style description applies at both the cell and tissue levels, we show how geometric factors play an increasingly important role at larger scales. In particular, we present and exploit measurements of cell-wall lengths and thicknesses in characterising mechanical properties of the whole tissue. Our model demonstrates the geometric advantage possessed by epidermal cells, relative to other cell layers, in influencing elongation and bending properties, which we quantify for the *Arabidopsis* root. The model also reveals a fundamental relationship between RER and curvature growth rate, providing new insights into existing observations (Chavarría-Krauser *et al.* 2008), which we exploit to derive predictions of gravitropic bending angles.

## Materials and Methods

### Plant material and growth conditions

All lines used in this study were in the *Arabidopsis thaliana* (L.) Heynh. Columbia-0 background (Col-0). Seeds were surface-sterilized and sown on vertical 125 × 125 mm square Petri plates as detailed previously (De Rybel *et al.* 2010). Each plate contained 60 ml 1/2 strength Murashige and Skoog media (Sigma) solidified with 1% (w/v) agar. After 2 d at 4°C, plates were transferred to controlled-environment chambers at 23°C under continuous light at a photon flux density of 150 μmol m^**−**2^ s^**−**1^.

### Pressure probe measurements

Seven-d-old plants were transferred to a fresh growth plate and mounted vertically on an adapted light microscope (Axiostar; Carl Zeiss Ltd, Welwyn Garden City, UK). Filamented borosilicate glass microcapillaries (Harvard Apparatus Ltd, Edenbridge, UK) were pulled using a Flaming/Brown puller (Model P-97; Sutter Instrument Co., Novarto, USA) to produce micropipettes with an external tip diameter of 1 μm. Micropipettes were filled with silicone oil (polydimethylsiloxane viscosity standard; Brookfield Engineering Laboratories Inc., Middleboro, MA, USA) and connected to a custom-built cell pressure probe (details available at: http://www.cpib.ac.uk/tools-resources/pressure-probe). Turgor was measured in individual cells with the micropipette position along the longitudinal axis of the root determined from video recordings of each impalement. A successful recording was taken to be one in which the oil/sap meniscus was repositioned during measurement with turgor values remaining similar before and after repositioning, indicating an unblocked tip. Pressure measurements were recorded at 30 Hz via a data acquisition board (PCI-6221; National Instruments Corp. (NI), Austin, USA) and analysed using custom-written LabView software (NI, http://www.cpib.ac.uk/tools-resources/equipment-software/).

### Root cross-section geometries

Seven-d-old plants expressing a fluorescent plasma membrane marker (GFP-Lti6A, Cutler *et al.* 2000) were imaged using a confocal laser scanning microscope (Leica SP5; Leica Microsystems, Wetzlar, Germany). Z-stack image sets were loaded in Fiji (http://fiji.sc/Fiji) using the LOCI BioFormats image importer plugin. Cross-section images were then generated using the Volume Viewer plugin (http://fiji.sc/Volume_Viewer), ensuring that each cross-section was orthogonal to the longitudinal axis of the root. This cross-sectional image was then calibrated with the correct pixel heights and widths in μm, and measurements were taken of the required wall lengths using Fiji's measuring tools.

### Transmission electron microscopy (TEM) cell-wall measurements

Seven-d-old roots were fixed in 0. 1 M cacodylate buffer (pH 7) containing 2% paraformaldehyde (Sigma) and 1% glutaraldehyde (TAAB) for 16 h at 4°C. The tissue was thoroughly rinsed in 0.1 M cacodylate buffer (TAAB) and postfixed in 1% osmium tetroxide (Sigma) for 2 h at room temperature. After fixation, samples were washed in 0.1 M cacodylate buffer, dehydrated in an ethanol series, embedded in LR White resin (TAAB) and polymerized at 60°C. Ultrathin sections (50–70 nm) were cut with a diamond knife on a Leica EM UC6 ultramicrotome and collected on copper grids (Formvar/carbon coated, 2 × 1 mm slot copper grids, Agar Scientific, Stansted, Essex, UK). Sections were double stained with saturated uranyl acetate in 50% (v/v) ethanol and Reynolds lead citrate and examined under a FEI Tecnai 12 BioTWIN transmission electron microscope.

**Figure 1:**
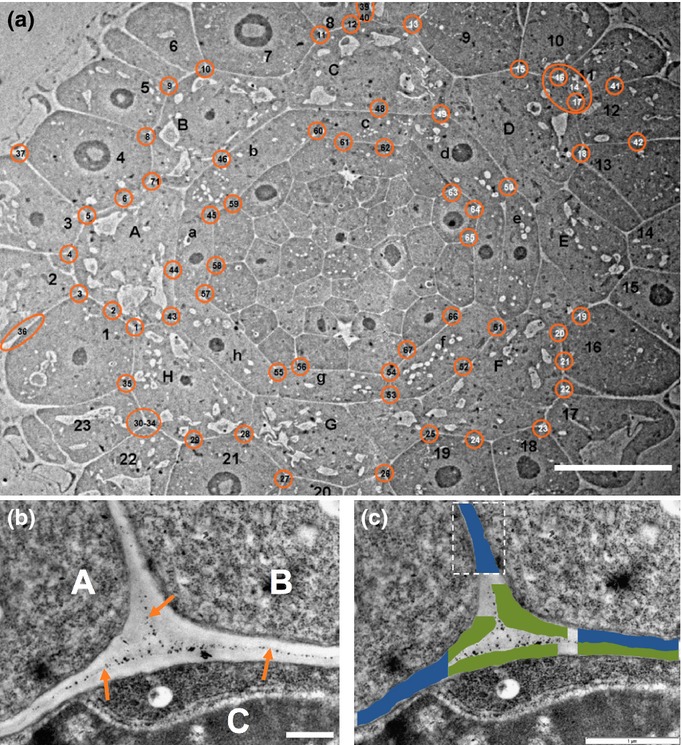
(a) Transmission electron micrograph of a section through an *Arabidopsis* root tip. 1–23, epidermis; A–H, cortex; a–h, endodermis. Regions circled in orange were imaged at higher resolution and used in subsequent determinations of cell-wall thicknesses. Bar, 20 μm. (b) Micrograph of region 25 in (a). A, B, cortical cells; C, epidermal cell; orange arrows, position of the middle lamella; bar, 500 nm. (c) Cell-wall silhouettes used for wall thickness quantification overlaid on image (b). Silhouettes in green represent single walls at junctions, those in blue represent two walls. Bar, 1 μm.

Transmission electron microscopy (TEM) images of transverse sections of five sample roots were prepared from two zones of the developing root tip: the meristematic zone (labelled 1A, 150–250 μm from the root tip) and the late meristematic zone (labelled 1B, 350–450 μm from the root tip). Technical limitations prevented measurements being taken further along the root; obtaining more precise wall thickness measurements along the length of the EZ is an area for future study. For each of the TEM images, regions between cell types of interest (epidermis, cortex, endodermis and pericycle) were identified, with the exception of the outer epidermal walls in Zone 1B which were too indistinct to be confidently identified. An example TEM image with wall regions of interest identified in orange is shown in Fig. 1(a). Each region of interest was then imaged at higher magnification (for example, Fig. 1b) to allow quantification of cell-wall thickness. To extract wall locations the individual micrographs were loaded into Photoshop (Adobe, San Jose, CA, USA). The magnetic lasso tool was used to semi-automatically select the region of the image around the cell wall. The cell wall selection was then converted to a binary silhouette, and that image cropped to fit around the selection. The resulting cropped image (see Fig. 1c) guaranteed that the wall region extended out of the image on at least two borders. The edges of the wall region were then transformed into lists of *x−y* Cartesian coordinates in the image plane (one per pixel). The distance between these wall ‘sides’ was then determined. For each pixel coordinate in one edge, the nearest pixel in the opposing edge was determined and the Euclidean distance between these two points calculated. This resulted in several hundred thickness measurements for each imaged section of wall, which we summarise with a single mean value per silhouette. The walls were classified according to the cells on either side of the wall. In the case of walls between two cells, the thickness of the wall between each cell and the middle lamella was determined. The data for each cell type show a lognormal distribution. We therefore take logarithms of the data and calculate the mean and standard error before exponentiating to calculate asymmetric confidence intervals. The presence of lateral root cap cells necessitated manual measurement of wall thicknesses for the outer epidermal cell walls. Wall thicknesses of 21 cells from four roots were manually measured in multiple positions using Fiji software described previously.

### Mathematical model: upscaling from cell to tissue

We developed a mathematical model (explained fully in Supporting Information Notes S1, S2) to determine the contribution of individual cell walls to the biomechanical properties of the whole tissue. We assume that turgor pressure drives growth, at a rate that is determined by the properties of cell walls (see the Results section for experimental justification). We relate the rate of cell elongation to the rate of curvature generation and demonstrate how gravitropic bending angle is related to asymmetries in the biomechanical properties of the root cross-section.

**Figure 2:**
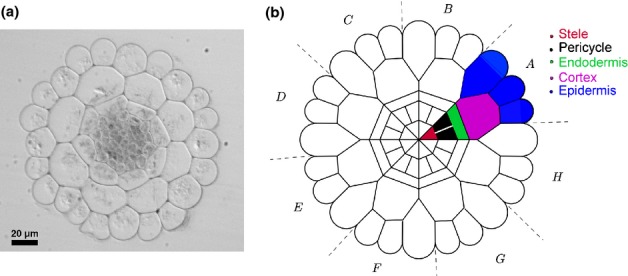
(a) An image of an *Arabidopsis* root cross-section. (b) Idealised root cross-section. Axial cell walls are represented as polygons and turgor pressure is assumed to be uniform across the root, so that only epidermal cells have curved walls. We label the ‘tiles’ of the tessellation (the eight cells in the repeating unit) A–H. See Supporting Information Notes S2 and Figs S2, S3.

We used confocal images of real root cross-sections to create an idealised template of the cell-wall network (Figs 2, S1). Because the mechanical anisotropy of cell walls resists radial expansion (Dyson & Jensen, 2010), we assume that the template of a given material cross-section is conserved during elongation. For simplicity, we adopt a quasi-plane-strain approximation, assuming that the entire root cross-section undergoes uniform axial strain (in elongation) or a nearly uniform strain (in bending, allowing for a weak linear strain gradient across the cross-section). However, we allow for the possibility of material properties varying between different cells.

We use the Lockhart equation (Lockhart, 1965; see Eqn S2) to model the elongation of each cell wall segment; this constitutive assumption is relatively simple to implement but is sufficient to describe elongation and gentle bending, and is supported by a micromechanical model of the cell wall (Dyson *et al.* 2012). It accounts for irreversible (viscous) deformation of the root; we do not consider reversible (elastic) deformations in the present study. The Lockhart equation provides predictions of kinematic quantities (such as the RER) in terms of material properties of the root.

We assign to each cell-wall segment within the cross-section template a wall extensibility ϕ [kg^**−**1^ s] and a wall yield stress resultant *Y* [kg s^**−**2^]. These can be related to the intrinsic extensional matrix viscosity μ [kg m^**−**1^ s^**−**1^] and yield stress *y* [kg m^**−**1^ s^**−**2^] of an individual cell-wall's material using 
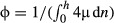
 (Dyson & Jensen, 2010) and 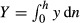 (*n*, measured distance through each cell wall; *h*, thickness of the composite cell-wall segment). Our model accounts for the possibility that μ and *y* may vary from cell to cell, leading to a step change within the composite cell wall. Thus the RER (or axial strain rate) of an element of individual cell wall satisfies RER_cell_ = ϕ (*T* − *Y*) provided *T *> *Y* (*T*, axial tension in the wall); in pure elongation, all walls in the same cross-section share the same RER but may have different tensions as a result of differing mechanical properties, allowing for gradients of tissue stress across the cross-section (Baskin & Jensen, 2013). We then evaluate line integrals (denoted with 〈 · 〉) over the entire network of cell walls in the cross-section, defining the effective tissue extensibility and tissue yield force to be, respectively:Eqn 1
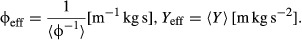


We compute these integrals explicitly using geometric data from the idealised cross-section (Fig. 2b), incorporating wall thickness measurements as described above. (Thus if *Y*, say, is uniform along a given cell wall of length *l*, then this will contribute *Yl* to the integral 〈*Y*〉).

Assuming that growth is not hindered by the external environment (considering, for example, a seedling growing on an agar plate instead of in a stiff soil), then across any cross-section of the root the net axial force and the net moment of axial force must both vanish. Because only the outer epidermal walls are appreciably curved in the image of a root cross-section (Fig. 2a), we may assume that turgor pressure is uniform across a given cross-section. (This assumption can be relaxed, as explained in Notes S1.) Thus the turgor pressure *P*, acting over the root's cross-sectional area A, is balanced by the axial tension *T* integrated along all the walls of the cross-section, 〈*T*〉, according to *P*A = 〈*T*〉. Neglecting any elastic deformations, we assume that no elongation takes place if *P* is sufficiently low. However, as *P* increases, walls yield sequentially (without the root elongating) with *T *≤ *Y* in each wall, until *P*A = 〈*Y*〉; once all walls have yielded, the root can elongate.

As explained in Notes S1, the RER for a segment of the root (tracking the growth of a slice of the root as it moves through the EZ) in terms of its effective tissue extensibility and tissue yield force is then:Eqn 2
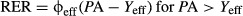


Here we have scaled up the Lockhart equation for an individual cell wall to its analogue (Eqn 2) for the whole root cross-section. The contributions of individual cell layers to the effective tissue extensibility and yield parameters arise from the line integrals (Eqn 1). The generation of curvature, *κ* (assumed small), is represented through the curvature generation rate (CGR) ≡ d*κ*/d*t* at a given root cross-section, which satisfiesEqn 3
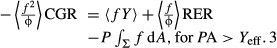


Here *f* measures the perpendicular distance to the cross-sectional diameter that is parallel to the axis of curvature. A further condition (see (S9)) is used to identify the direction of this axis within the root cross-section. In Eqn 3, the first and second terms on the right-hand side represent curvature generation by asymmetries in the yield and extensibility across the cross-section, respectively, whilst the third term represents curvature generation by geometric asymmetries in the cross-section (should they exist). A consequence of the Lockhart relation is that asymmetries in extensibility can generate curvature at a rate proportional to the RER, unlike asymmetries in yield.

### Mathematical model: predicting bending angle

Eqns 2 and 3 give the rate of stretching and the rate of curvature growth of slices of root material as they transit the EZ. These descriptions can be translated into distributions of length and curvature along the root, as explained in Band *et al.* (2012b), by describing the motion of individual cells along the cross-section in terms of continuous variables that depend on root-centreline arc-length *s* (measured from the root tip) and time *t*. This is illustrated with a deliberately simple example, chosen for clarity of analysis. Suppose that ϕ_eff_ and *Y*_eff_ are functions only of *s* (where the EZ lies in *s*_0_ < *s *< *s*_0_ + *s*_1_, say), making the RER a prescribed function of *s* but not time. Suppose also that the root centreline always lies within a single plane. Then, assuming that cells enter the EZ at a steady rate 1/*c* (one cell enters and one cell leaves the EZ in a time interval *c*), the cell length *l*(*s*) (averaged across the cross-section) and the curvature distribution *κ*(*s*,*t*) along the centreline satisfyEqn 4a, b
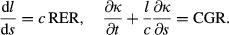


For the precise relationship between *l*(*s*) and the local cell length at location *s* see Band *et al.* (2012b). The quantity *v *= *l* /*c* is the speed at which cells move axially with respect to the root tip, so that d*v*/d*s *= RER; the material derivative in (Eqn 4b) represents the rate of change in the frame of a moving cell (Chavarría-Krauser, 2006). We suppose that cells enter the EZ at length *l*_0_ and leave it at length β*l*_0_ where β ≫ 1, so that *V *= β*l*_0_/*c* is speed of the root tip relative to the mature zone. Equivalently, in terms of characteristics, (4b) may be expressed asEqn 5
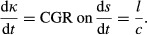


We then assume that a bend is generated in a growing root in response to a localised signal (such as auxin) that travels shootwards from the root tip. Suppose, for example, that curvature is generated solely by transverse gradients in extensibility (so that only the second term on the right-hand side of Eqn 3 is significant). To illustrate, we assume that the curvature generation factor 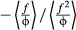 has magnitude *A*_0_ over an interval *t*_*a*_ for any fixed *s* in the EZ, being zero otherwise, and that this pulse travels shootwards at a uniform speed *V*, matching the speed at which cells leave the EZ without disrupting the root's steady elongation. Combining (3–5), the curvature acquired by the cells leaving the EZ which have been exposed to the signal within the EZ can be written in the formEqn 6
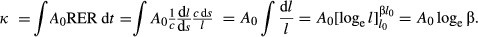


Thus, the large relative increase in cell length over the EZ gives rise to a moderate increase in curvature (noting that for cells that expand 30-fold in length log_e_ β* *≈ 3.4). We emphasise that this simple expression is used to illustrate the likely magnitude of curvature generated by a transverse gradient of extensibility, and could be modified to incorporate variations in *A*_0_ as the pulse of asymmetric extensibility travels along the root. A more rigorous statement of this result (accounting for cells which are exposed to the bending signal for only part of the EZ) is given in Notes S3. Then, once the signal has passed through the EZ, a line of cells of distance *Vt*_*a*_ ≡ (β*l*_0_/*c*)*t*_*a*_ will have been exposed to the signal. The angle Δθ through which the root bends (see Fig. S1) is determined by integrating the curvature over this distance, yieldingEqn 7
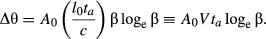


This angle is independent of the specific distribution of RER across the EZ, as demonstrated in Notes S3; an additional contribution to the bending angle may arise from asymmetries in yield, as discussed below.

## Results

### Pressure probe measurements show turgor to be uniform along the EZ

**Figure 3:**
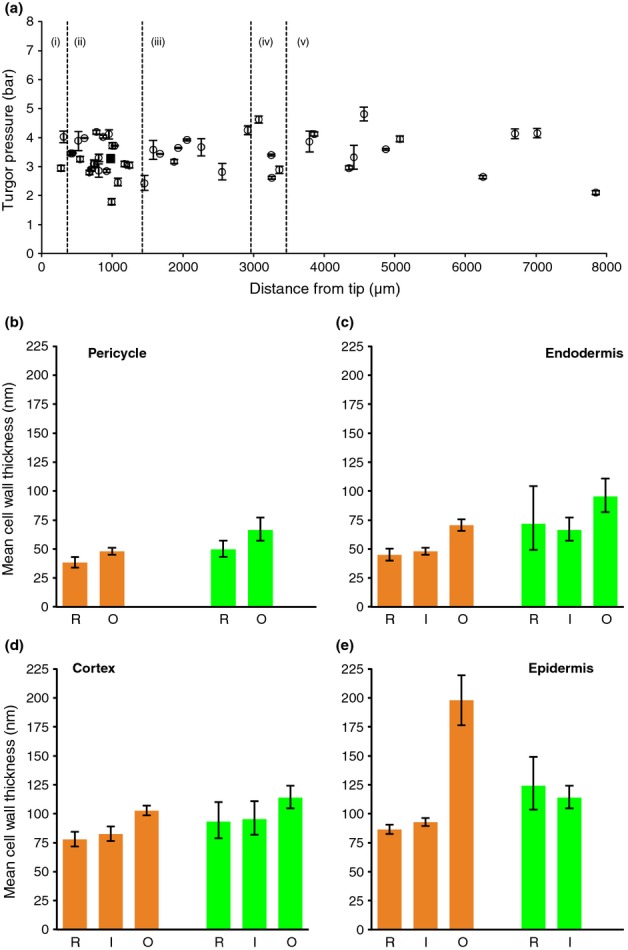
(a) Turgor pressure (*P*) measurement in *Arabidopsis* epidermal cells along the longitudinal root axis. Dashed lines represent the boundaries of the five developmental zones as defined in De Rybel *et al*. (2010): (i) meristem, (ii) accelerating elongation zone, (iii) decelerating elongation zone, (iv) mature zone and (v) reference zone. Error bars, ± 1 SD. (b–e) Measurements of cell-wall thicknesses in *Arabidopsis* for Z1A (early meristem, orange) and Z1B (late meristem, green). R, radial walls; I, inner walls; O, outer walls for (b) pericycle, (c) endodermis, (d) cortex and (e) epidermis. Error bars, ± 2 SE.

In principle, both turgor and cell-wall properties may independently regulate tissue elongation rates. To assess the role of pressure regulation, we constructed a cell pressure probe (see the Materials and Methods section) to measure the turgor of individual epidermal cells in the *Arabidopsis* root. Epidermal cells transit a series of zones during their development that we define as the meristem, accelerating EZ, decelerating EZ, mature zone and reference zone (rootward to shootward, respectively). As epidermal cells within the meristem are experimentally inaccessible due to the overlaying lateral root cap, we sampled cells using the cell pressure probe across the last four of these five developmental zones, spanning *c*. 7.5 mm from the tip (Fig. 3a). See Notes S4 and Table S1 for data. Our measurements revealed that turgor pressure in *Arabidopsis* epidermal cells effectively remained constant in the four sampled developmental zones along the longitudinal axis of the root, as has been previously reported in epidermal cells of wheat roots (Pritchard *et al.* 1987) and maize cortical cells (Spollen & Sharp, 1991; Pritchard *et al.* 1993; Shimazaki *et al.* 2005), and consistent with simulations suggesting rapid osmoregulation (Chavarría-Krauser *et al.* 2005). We conclude that axial turgor pressure gradients are not significant in regulating growth rates along the root.

### TEM reveals that cell-wall thicknesses vary between different tissues

A key parameter governing the contribution of the different cells to the overall growth is the distribution of cell-wall thickness across the root cross-section. To accurately characterize thicknesses in our model, we imaged cell walls using TEM (Fig. 1). Analysing these TEM images, we characterised cell-wall thicknesses at the various cell–cell junctions in the *Arabidopsis* root, as shown in Fig. 3(b–e). In cross-section, the walls of individual cells can be grouped into three classes: radial walls between cells of the same type (R in Fig. 3b–e) and the inner and outer tangential walls between the various tissue layers (I and O, respectively in Fig. 3b–e). In the endodermis, cortex and epidermis no significant differences were found between the thickness of the radial walls and the inner tangential walls of the same tissue in each of the zones measured. In all tissues, the mean thickness of the outer tangential wall was greater than that of the inner and radial walls, suggesting that turgor may be elevated towards the axis of the root. The outer wall of the epidermis was substantially thicker than all other walls measured (Fig. 3e). *t*-tests on the logarithmic data reveal that in zone Z1A (early meristem), the outer walls of cortical cells are thicker than the adjoining inner epidermal walls (*P *=* *0.0003), and similarly the inner cortical walls are thicker than outer endodermal walls (*P *=* *0.003). See Notes S5 and Table S2 for further details. Technical limitations prevented measurement of cell-wall thicknesses of segments surrounding the central stele cells. We therefore use the minimum value measured (that of the internal pericycle walls) for these cells. Our measurements revealed that cell-wall thicknesses increase from the pericycle to the epidermis, and provided detailed parameters to use in our mathematical model.

### Modelling reveals the predominant influence of the epidermis in controlling root elongation and curvature generation

**Figure 4:**
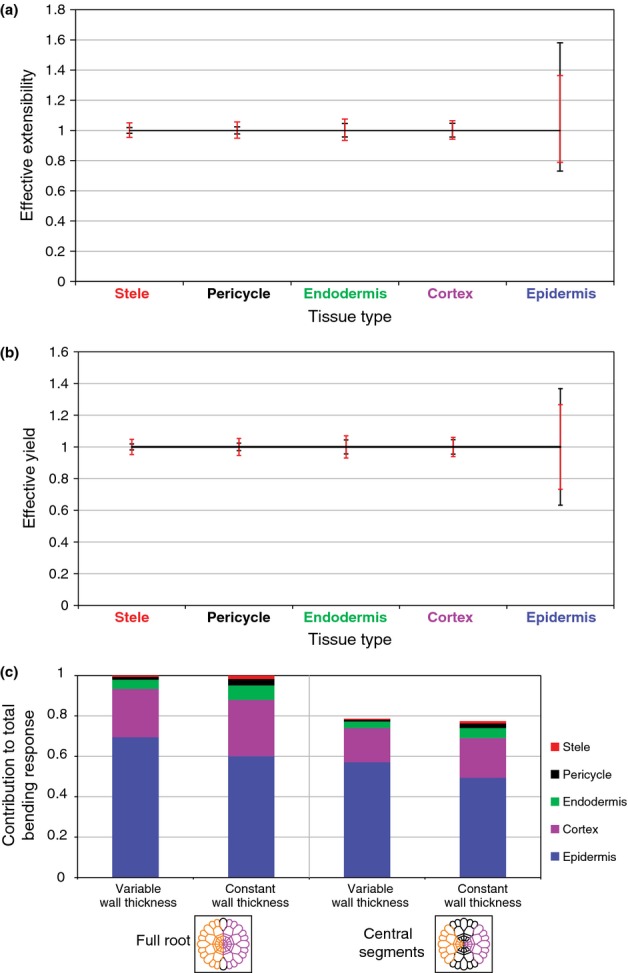
(a) Predicted changes in tissue extensibility (ϕ_eff_) (relative to baseline value of unity) due to ± 50% changes in wall viscosity (μ) of the five individual cell layers. (b) Predicted changes in tissue yield force (*Y*_eff_) (relative to baseline value of unity) due to changes in yield stress *y* of individual cell layers of ± 50%. Black lines give the effect when differences in wall thicknesses are incorporated, red lines when they are neglected. (c) Predicted relative contributions of different cell layers to the terms contributing to growth of curvature arising from asymmetries in extensibility (see 〈*f*/ϕ〉 in Eqn 3), with and without variations in cell wall thickness. Cartoons illustrate two different patterns of asymmetry considered. The measured root geometries and cell-wall thicknesses in *Arabidopsis* are as described in Figs2 and 3, where the mechanical properties are scaled to make the base extensibility, yield and curvature generation unity.

According to our model, the elongation of the root is determined by the effective tissue extensibility ϕ_eff_ and effective tissue yield *Y*_eff_. This relationship is captured in Eqn 2 (see Materials and Methods section), which is an ‘upscaled’ version of the Lockhart equation satisfied by individual cell walls. At the tissue scale, geometry plays a significant role in determining the biomechanical parameters, as determined by Eqn 1. Specifically, cell layers towards the periphery of the root, having larger net perimeter, contribute more to the line integrals in Eqn 1 than cells in inner tissue layers. We take the data from the idealised root section geometries and cell-wall thicknesses described above to parameterise the model. We use thickness values from the late meristematic zone, as thickening of the cell walls as the cells progress through the meristem is observed (see Notes S5). We note, however, that the model predictions are relatively insensitive to the thickness measurements; including variations in the root cross-section geometry alone produces the majority of the response (Fig. 4).

In order to quantify the contributions of individual cell files to the effective extensibility of the root cross-section, we perturbed the viscosity μ (and hence the extensibility) of individual cell files by ± 50% in our model. The predicted change in ϕ_eff_ (illustrated in Fig. 4a) shows the greatest sensitivity to the properties of the epidermis. This is consistent with the fact that epidermal cell walls have the greatest net length in the cross-section (Fig. 2a) and explains how auxin, which targets the epidermis (Swarup *et al.* 2005), can have strong control over elongation rates. Likewise, *Y*_eff_ is also predicted to be most sensitive to the properties of the epidermis (Fig. 4b). Thus, hormones that target internal layers, such as gibberellin which targets the endodermis (Úbeda-Tomás *et al.*, 2008), can inhibit growth only by inducing proportionately larger changes in cell-wall properties (although a small change in *Y*_eff_, sufficient to take it across the threshold *P*A, can have a large effect on elongation). The reduction in ϕ_eff_ due to stiffening the epidermis (decreasing its μ by 50%) is smaller than the increase in ϕ_eff_ due to softening the epidermis by the same proportion. This is because of the nonlinear relationship between ϕ and ϕ_eff_ in Eqn 1, in comparison to the linear relationship between Y and *Y*_eff_.

In order to estimate the relative contribution of different cell files to the generation of curvature, we prescribed anti-symmetric perturbations in the extensibility of each cell's walls (such that additions to one side of the root cross-section were exactly compensated for by reductions on the opposite of the cross-section) (Fig. 4c). The predicted effect on the normalised CGR again shows greatest sensitivity to the epidermis, with the majority of the bending response being produced when only the epidermal layer is perturbed. Similarly, when only the ‘central segments’ (i.e. the ‘tiles’ H, A, D, E, in Fig. 2b) are perturbed, the majority of the bending response is predicted to be determined by the epidermis.

### Cell expansion amplifies curvature generation

Eqn 3 demonstrates how curvature is generated by differential growth. Within the EZ, gradients across the root in extensibility, yield and geometry can all induce bending. To illustrate this, we considered only a transverse gradient in extensibility. In this case, the CGR is proportional to the RER, by a factor 
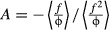
 (with dimensions of inverse length) which incorporates geometric information and the distribution of extensibility across different cell layers. The relative contributions of the different cell layers to *A* are illustrated in Fig. 4(c).

In order to explore the consequences of the relationship between CGR and RER, we examined a simple example in which elongation proceeds steadily while a wave of asymmetric softening passes along the root (mimicking measured shootward-propagating waves of curvature (Durham Brooks *et al.* 2010)). If it is assumed that *A* takes the constant value *A*_0_ within the wave, then the curvature accumulated by cells as they pass through the EZ has magnitude *A*_0_log_e_β (see Eqn 6>), where β is the factor by which cells elongate as they cross the EZ. According to this simple model, the accumulated curvature is independent of the precise distribution of RER across the EZ. However, this factor would be reduced if, for example, dilution of a signal due to cell elongation were to allow *A* to fall as cells grow.

While the accumulation of curvature may appear modest, the angle through which the root bends is determined by an integral of curvature along the root. Thus, any factors that increase the exposure of cells to softening will enhance bending. We illustrate this with two simple examples. First, suppose that cells entering the EZ are exposed to an asymmetric softening signal over a time interval *t*_*a*_, but that the signal is not transported between neighbouring cells. Then on leaving the EZ, cells are moving at a speed *V* ≡ β*l*_0_/*c* and a length of root *Vt*_*a*_ has been exposed to the signal, leading to a total bending angle given by Eqn 7. Alternatively, suppose that the signal propagates along the root at a steady speed; a natural speed to consider in this example is *V*, the speed at which mature cells leave the EZ, requiring active cell-to-cell transport only where cells are shorter than β*l*_0_. In this case also, a length of root *Vt*_*a*_ is exposed to the signal, again leading to bending at an angle (Eqn 7; as explained in detail in Notes S3 and Fig. S4). These two examples differ in the timing of the response relative to the signal (with active transport allowing a quicker response), but share the same net bending angle.

The bending angle, being a dimensionless quantity, depends on ratios of lengths and ratios of times in Eqn 7. The exposure time *t*_*a*_ appears in the ratio *t*_*a*_/*c*, where *c* is the time at which cells enter or leave the EZ. Thus, the bending angle is linearly proportional to *t*_*a*_ but can also be manipulated by changes in *c*. The curvature factor *A*_0_ appears in the dimensionless product *A*_0 _*l*_0_, where *l*_0_ is the length of a cell entering the EZ. The dependence of bending angle on cell elongation factor β is nonlinear: the factor log_e_β arises from accumulation of curvature across the EZ while the additional factor β reflects magnification of the signal by cell elongation.

When transverse gradients in extensibility are small (differences across the cross-section are small compared with the mean, 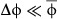), then we can approximate *A* as 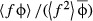 (see Notes S3), which we may estimate as 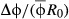 (*R*_0_, root radius). Further, if the duration of the bending signal is comparable to the transit time through the EZ, it follows from Eqn 7 and Eqn S55 in Notes S3 that in order to generate an appreciable bending angle the relative difference in extensibility across the root need be only of magnitude (*R*_0_/*s*_1_)/log_e_β≲ 1% (taking *R*_0_* *=* *50 μm, *s*_1_* *=* *2500 μm; *s*_1_, length of the EZ). Thus, while the epidermis has a three-fold advantage over the cortex in generating a bending response (Fig. 4c), the model predicts that the relative difference in extensibility across the cortex need only be 3% in order to generate significant bending. Alternatively, this enhancement could be provided by extending the duration of the bending signal to the cortex proportionately.

Finally, the model identifies the angle *θ*_0_ = *A*_0_*s*_1_Glog_e_β (see Eqn S79) as a significant quantity in the gravitropic control mechanism. Here G is a constant between zero and unity depending on the precise form of the RER (which is readily computed from kinematic data). If it is assumed that the bending signal is carried by cells as they move through the EZ, and that the duration of the signal *t*_*a*_ exceeds the time for a cell to traverse the EZ, then *θ*_*0*_ represents the component of the net bending angle turned by the root after the bending signal has been terminated at the root tip. As with the net bending angle (Eqn 7), the contributions of individual cell layers to this residual bending angle appear through the linear factor *A*_0_.

## Discussion

The concept of the epidermis taking a dominant role in controlling the elongation rate of plant organs is well established, particularly given the observation of inner tissues of a stem elongating when the outer layers are removed (Kutschera & Niklas, 2007). This demonstrates that, *in situ*, the inner tissues are under net compression and the outer layers under tension, with the epidermis appearing to restrain rapid growth. In aerial organs, the associated gradients of tissue stress (the stress field averaged over multiple cells, but not necessarily the whole cross-section) may contribute to the organ's structural stability (Vandiver & Goriely, 2008). The gradient of tissue stress can be explained through gradients in material properties, although active stress generation is also a candidate mechanism (Baskin & Jensen, 2013). In root systems, however, and *Arabidopsis* in particular, it is less clear that substantial gradients in tissue stress exist across a cross-section. However, as our model demonstrates, the epidermis still maintains its predominant role in regulating elongation.

There is an intuitive explanation for this observation: epidermal cells have much greater net perimeter in the root cross-section than any other cell layer. To quantify this advantage, we took detailed measurements of cell-wall lengths and thicknesses across a set of cross-sections. We adopted the widely accepted Lockhart model for cell and tissue elongation, and derived the relationship between tissue-level mechanical parameters (yield and extensibility) in terms of properties of individual cells. Incorporating geometric data, we showed how the epidermis has at least a six-fold influence compared with any internal layer in determining tissue-level growth parameters ϕ_eff_ and *Y*_eff_ (Fig. 4a,b). At present, however, the contributions of these individual parameters to patterns of root elongation across the EZ remain a matter of debate: the initiation of growth at the distal end of the EZ can be expected to arise through a drop in yield, given our measurements showing that turgor is uniform along the root (Fig. 3a); however, the inhibition of growth at the proximal end can be explained either by an increase in effective yield, or a substantial drop in effective extensibility (possibly associated with reorientation of microfibrils in highly elongated cells (Dyson & Jensen, 2010)).

Naturally, asymmetries across the root can generate curvature. As shown in Eqn 3, three independent mechanisms emerge: a gradient of yield across the cross-section; a gradient of extensibility; and material asymmetry. The last of these may always be present to some extent, and the effect has been recognised previously in simulations (Fozard *et al.* 2013); however, in normal roots it is likely to be compensated by tropic responses (or possibly a mechanism of proprioception (Bastien *et al.* 2013)), so we do not consider it further. Geometrical factors play a crucial role in determining the relative magnitudes of the remaining contributions to the CGR and in determining the net direction of bending, with the epidermis once more taking a dominant role (Fig. 4c).

According to our simple model, the tension in a cell wall is *T *= *Y*  + (RER/ϕ) when an organ is elongating (with RER > 0). Thus, the tension in a peripheral cell layer may be elevated relative to inner tissues by having larger *Y* or smaller ϕ, leading to gradients of tissue stress. During bending, the tissue stress is inherently asymmetric, although the total stress and its moment must vanish when integrated across the root cross-section (in the absence of external forces).

Significantly, the component of CGR in Eqn 3 generated by ϕ is proportional to the RER, unlike that arising from yield or geometry. Fortunately, distributions of CGR and RER have previously been measured along *Arabidopsis* roots during gravitropic bending by Chavarría-Krauser *et al*. (2008). (Note that the curvature growth rate reported by Chavarría-Krauser *et al*. (2008) is the spatial gradient of a Lagrangian time derivative of root angle; this differs from the Lagrangian time derivative of a spatial gradient of root angle used here. We assume the difference may be neglected in the argument that follows.) Their data show that, 3 or more hours after the gravity stimulus in the central EZ, the CGR and RER have similar distributions, with closely aligned maxima, supporting the hypothesis that ϕ generates curvature in this region. However, within the first hour after the stimulus, in the distal EZ, the CGR is large where the RER is small (Chavarría-Krauser *et al.* 2008), making it more plausible that *Y*, rather than ϕ, generates curvature in this region. Significantly, curvature generation in the distal EZ was observed both in wild-type and in a *pin3* auxin transport mutant, whereas that in the central EZ was seen in wild-type but not the mutant. PIN3 is thought to be a key player in creating the asymmetric auxin fluxes from the root tip during a gravitropic response, therefore these data suggest that the PIN3-dependent auxin asymmetry generates the curvature in the central EZ but not in the distal EZ (whether the curvature in the distal EZ is due to auxin asymmetries created by a different process, or a different mechanism entirely, remains unresolved).

A potential explanation of this observation is that distinct structural elements of the cell wall are targeted in the two regions. In previous models, we have shown how yield can arise from the action of hemicellulose crosslinks (Dyson *et al.* 2012), while extensibility can be characterised primarily by properties of the pectin matrix (Dyson & Jensen, 2010). Thus, we can hypothesise that the PIN3-dependent auxin asymmetry regulates the matrix, but that the pathway targeting crosslinks is PIN3-independent.

Given evidence that extensibility gradients can generate curvature, we computed the bending angle arising from a constant transverse gradient of extensibility propagating along the root. Because CGR is then proportional to RER, the curvature acquired by cells as they move through the EZ is proportional to log_e_β (see Eqn 6), where β is the factor by which cells elongate through the EZ. The bending angle is then determined by the length of root exposed to the extensibility gradient, which in our model was expressed as the speed *V* at which the cells leave the EZ times a time of exposure *t*_*a*_, giving the simple expression (Eqn 7). Remarkably, the predicted net bending angle is insensitive to patterns of growth within the EZ, depending only on β. This prediction neglects additional bending driven by gradients of yield, and takes no account of the gravitropic control system that initiates the bending response, factors which will be addressed in future studies.

There are numerous additional weaknesses in our modelling approach that remain to be addressed: the validity of the underlying Lockhart model (which can be replaced with much more sophisticated constitutive assumptions (Huang *et al.* 2012), and by employing simulations that capture the anisotropic viscoelastic properties of individual cells (Fozard *et al.* 2013)); the assumption of quasi-stationarity (noting for example that the RER distribution may change after a gravitropic stimulus (Chavarría-Krauser *et al.* 2008)); the neglect of environmental forces that enter the force and moment balances that we employed; the incorporation of cell division (Chavarría-Krauser & Schurr, 2004); and three-dimensional effects such as twisting and torsion.

In summary, we have shown how, for a highly organised tissue such as the primary root of *Arabidopsis*, cell-level properties can be integrated to determine properties at the tissue level. Our approach provides an efficient strategy to incorporate the properties of individual cell walls in multiscale models for root gravitropism. Our model predicts that the parameters determining root elongation and curvature generation are most sensitive to the material properties of the epidermis, which is targeted by auxin (Swarup *et al.* 2005). Hormones targeting internal layers must exert a greater influence on wall mechanical properties in order to influence growth and curvature rates. We have shown how geometric data can be used to quantify this difference, and demonstrated how to predict resulting bending angles.

## Supporting Information

Fig S1 A typical root showing the cross-section, cell wall network and axes. **Fig. S2 **An idealised root cross-section. **Fig. S3 **Geometric labels for a ‘slice’ of root. **Fig. S4** A space–time diagram for cells moving through the EZ. **Table S1 **Turgor pressure data as plotted in Fig. 3(a) **Table S2** Statistical significance of comparison of cell thicknesses Z1A vs Z1B **Notes S1 **Model description for upscaling cell wall properties to the tissue level. **Notes S2 **Model description for using an idealised root geometry. **Notes S3 **Model description for bending induced by asymmetric extensibility. **Notes S4** Turgor pressure data. **Notes S5 **Further cell wall thickness analysis.
