# Supplementary material for: Mechanical modelling quantifies the functional importance of outer tissue layers during root elongation and bending
Source: New Phytol. 2014 Mar 18;202(4):1212–22. doi: 10.1111/nph.12764 (PMC4286105; doi:10.1111/nph.12764)
Supplement: Fig S1 — A typical root showing the cross-section, cell wall network and axes. Fig. S2 An idealised root cross-section. Fig. S3 Geometric labels for a ‘slice’ of root. Fig. S4 A space–time diagram for cells moving through the EZ. Table S1 Turgor pressure data as plotted in Fig. 3(a) Table S2 Statistical significance of comparison of cell thicknesses Z1A vs Z1B Notes S1 Model description for upscaling cell wall properties to the tissue level. Notes S2 Model description for using an idealised root geometry. Notes S3 Model description for bending induced by asymmetric extensibility. Notes S4 Turgor pressure data. Notes S5 Further cell wall thickness analysis. [file nph0202-1212-SD1.pdf]

Mechanical modelling quantifies the functional importance of outer tissue layers during root elongation and bending.

Supporting Information Notes S1-S5, Figs S1-S4 and Tables S1-S2

R.J. Dyson, G. Vizcay-Barrena, L.R. Band, A.N. Fernandes, A.P. French, J.A. Fozard, T.C. Hodgman, K. Kenobi, T.P. Pridmore, M. Stout, D.M. Wells, M.H. Wilson, M.J. Bennett & O.E. Jensen

12th February 2014

Introduction

In this Supporting Information, we provide details of a simple three-dimensional model for a growing root, consisting of polyhedral cells in which each cell elongates according to the 'Lockhart' model. We perform this upscaling of properties from cell wall to tissue level by tracking the properties of a single representative root cross section (Notes S1). We then use data for an idealised root cross section to determine how the individual cell properties govern the behaviour of the composite structure (Notes S2). Having determined the influence of individual cell files on the generation of curvature, we investigate how this curvature translates into changes in the angle of the root during a gravitropic bend, assuming the asymmetry which generates the curvature maintains the bend in a single plane (Notes S3). Finally, we give the pressure probe data (Notes S4) and further details of the cell wall thickness measurements (Notes S5).

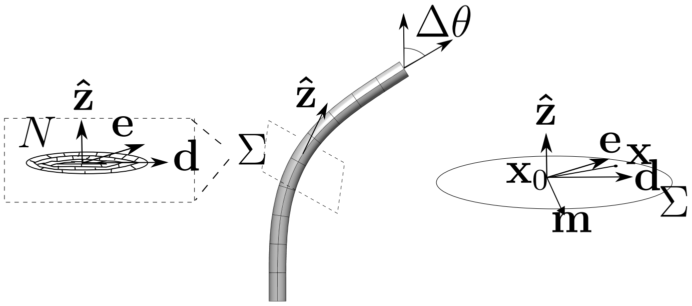

Figure S1: A typical root showing the cross-section, cell wall network and axes. Curvature is generated by differential expansion of cells either side of the root, creating a bending angle $\Delta\theta$.

Notes S1: Upscaling cell wall properties to tissue level

S1.1 Governing equations

The cells which form a root are tightly bound together through their individual cell walls, such that the individual walls of neighbouring cells may be considered to be a single composite wall segment, where each cell controls the mechanical properties of its half of the wall thickness. We therefore consider a network N of composite cell wall segments which intersect a root cross-section Σ perpendicularly (see Fig. S1 and Fig. 2 of the main text). Let $\mathcal{A} = \int_\Sigma \mathrm{d}A$ be the area of Σ and $\mathcal{L} = \int_N \mathrm{d}\zeta$ be the total length of cell wall intersecting Σ, and define the operator $\langle f \rangle \equiv \int_N f \,\mathrm{d}\zeta$, where ζ denotes arc-length along each separate cell wall segment (in the plane of N). Let $\hat{\mathbf{z}}$ be the unit normal to Σ, oriented along the axis of the root (pointing away from the root tip) and take $\mathbf{x}_0 = \langle \mathbf{x} \rangle / \mathcal{L}$ in Σ so that $\langle \mathbf{m} \rangle = \mathbf{0}$, where $\mathbf{m}(\mathbf{x}) \equiv (\mathbf{x} - \mathbf{x}_0) \times \hat{\mathbf{z}}$ for a given $\mathbf{x} \in N$ (see Fig. S1). Thus $\mathbf{x}_0$ gives the centroid of the network N. Let $\mathbf{d}$ be a unit vector in the plane of the cross section (to be determined), and let $\mathbf{e} = \hat{\mathbf{z}} \times \mathbf{d}$ such that $\mathbf{x} - \mathbf{x}_0 = g\mathbf{d} + f\mathbf{e}$, $\mathbf{m} = f\mathbf{d} - g\mathbf{e}$, where

$$f(\mathbf{x}) = \mathbf{e} \cdot (\mathbf{x} - \mathbf{x}_0), \quad g(\mathbf{x}) = \mathbf{d} \cdot (\mathbf{x} - \mathbf{x}_0), \tag{S1}$$

so that $\langle g \rangle = 0$, $\langle f \rangle = 0$. The orientation of $\mathbf{d}$ and $\mathbf{e}$ within Σ is explained below. For a root growing in a frictionless medium (*i.e.* not accounting for any forces from the surrounding agar or soil) we assume that the cells expand as independent units subject to the constraint that they remain tightly adhered to their neighbours. We allow for elongation and bending of the whole root without shear or torsion. Growth in the radial direction is taken to be negligible, restricted by the mechanical

properties of the cross walls (Dyson & Jensen 2010), so the cross section Σ and network N are therefore both assumed constant in time as the cross-section Σ transverses the elongation zone.

Each cell wall segment within N is characterized by an axial tension $T(\mathbf{x})\hat{\mathbf{z}}$, an extensibility $\phi(\mathbf{x})$ and a yield $Y(\mathbf{x})$. Let $0 \leq n \leq h(\mathbf{x})$ be a coordinate system running through the thickness h of each cell wall segment. Following Dyson & Jensen (2010), we take $\phi(\mathbf{x}) = 1/\int_0^{h(\mathbf{x})} 4\mu(n)\ \mathrm{d}n$, $Y(\mathbf{x}) = \int_0^{h(\mathbf{x})} y\ \mathrm{d}n$ where $\mu(n)$ gives the extensional viscosity and $y(n)$ gives the yield stress of the cell wall segment, allowing for variations in the material properties of individual cell walls. Each cell is assumed to be subject to turgor pressure P. The relative elongation rate (RER) of each wall segment satisfies the Lockhart equation,

$$\mathrm{RER}_{\text{cell}} = \begin{cases} \phi(T - Y) & \text{assuming } T \geq Y \\ 0 & \text{when } T < Y. \end{cases} \tag{S2}$$

A segment of root is described kinematically in terms of the rate of axial extension $\mathrm{RER}(t)$ and the curvature $\kappa(t)$ of its centreline. We assume the axis of curvature is parallel to $\mathbf{d}$ (to be determined) and the centre of curvature lies at $\mathbf{x}_0 - \mathbf{e}/\kappa$. We assume that the curvature is small such that $|\kappa f| \ll 1$ for all $\mathbf{x} \in N$. For a short material segment of root, bounded by planes perpendicular to the centreline, for which the centreline has length $\delta l_0(t)$, the axial length passing through a point $\mathbf{x}$ in Σ is $\delta l(\mathbf{x}, t) = \delta l_0(1 + \kappa f(\mathbf{x}))$. Note that $\mathrm{RER} = (\frac{d(\delta l_0)}{dt})/\delta l_0$. Temporal changes in $\delta l_0(t)$ and $\kappa(t)$ lead to changes in δl and hence in $\mathrm{RER}_{\text{cell}}$ via

$$\mathrm{RER}_{\text{cell}}(\mathbf{x}, t) = \frac{1}{\delta l}\frac{d(\delta l)}{dt} = \frac{\delta l_0 \frac{d\kappa}{dt} f + \frac{d(\delta l_0)}{dt}(1 + \kappa f)}{\delta l_0(1 + \kappa f)} = \mathrm{RER} + \frac{d\kappa}{dt} f + O((\kappa f)^2). \tag{S3}$$

A force balance over the root cross section gives $\overline{P}\mathcal{A} = \langle T \rangle$, where $\overline{P}\mathcal{A} = \int_\Sigma P \mathrm{d}A$, implying that the turgor driving cell elongation is balanced by the net tension integrated over all cell walls in N. We neglect any additional axial forces on Σ due to interactions between the root and its external environment. For sufficiently low $\overline{P}$, the root will not elongate because the induced tension T in any wall will be less than the local yield Y. As $\overline{P}$ increases, the wall tensions will increase until $T = Y$ in each wall. If Y varies between walls, the wall with the greatest Y (Y_{max}, say) will be the last to yield. Finally, once $\overline{P}\mathcal{A} \geq \langle Y \rangle$, the root elongates with (from (S2))

$$\overline{P}\mathcal{A} = \langle Y \rangle + \left\langle \frac{1}{\phi}\mathrm{RER}_{\text{cell}} \right\rangle.$$

To leading order in κf, for which $\mathrm{RER}_{\text{cell}} \approx \mathrm{RER}$, this implies

$$\mathrm{RER} = \frac{1}{\langle \phi^{-1} \rangle}(\overline{P}\mathcal{A} - \langle Y \rangle) \qquad (\overline{P}\mathcal{A} > \langle Y \rangle), \tag{S4}$$

giving the effective tissue extensibility $\phi_{\text{eff}} = 1/\langle\phi^{-1}\rangle$ and tissue yield force $Y_{\text{eff}} = \langle Y\rangle$, as reported in Eq. (1) of the main text. If P is uniform across the root then $\overline{P} = P$ in (S4).

Having established the leading-order elongation behaviour we now investigate how variations across the cross section can lead to bending of the root by considering the moment acting on the centreline. A moment balance over a cross-section about $\mathbf{x}_0$ gives $\langle T\mathbf{m}\rangle = \int_\Sigma P\mathbf{m}\,\mathrm{d}A$. Resolving in the $\mathbf{d}$ and $\mathbf{e}$ directions, we obtain

$$\left\langle fY + \frac{f}{\phi}\mathrm{RER}_{\text{cell}}\right\rangle = \int_\Sigma Pf\,\mathrm{d}A, \quad \left\langle gY + \frac{g}{\phi}\mathrm{RER}_{\text{cell}}\right\rangle = \int_\Sigma Pg\,\mathrm{d}A, \quad (\overline{P}\mathcal{A} > \langle Y\rangle). \tag{S5}$$

Using (S3), to leading order in κf, this implies

$$\langle fY\rangle + \langle f/\phi\rangle\mathrm{RER} + \langle f^2/\phi\rangle\mathrm{CGR} = \int_\Sigma Pf\,\mathrm{d}A, \tag{S6}$$

$$\langle gY\rangle + \langle g/\phi\rangle\mathrm{RER} + \langle gf/\phi\rangle\mathrm{CGR} = \int_\Sigma Pg\,\mathrm{d}A, \tag{S7}$$

where $\mathrm{CGR} = \mathrm{d}\kappa/\mathrm{d}t$ is the curvature generation rate for the root slice of interest. Equations (S6, S7) combine to determine the bending response of the root to variations in geometry or mechanical properties across the cross section (see Eq. (3) of the main text). Eliminating κ yields a compatibility condition that can be used to determine $\mathbf{d}$ (subject to an arbitrary choice of sign). Define

$$C(Y;\phi) \equiv \langle fY\rangle\langle gf/\phi\rangle - \langle gY\rangle\langle f^2/\phi\rangle. \tag{S8}$$

Then the condition is

$$C(Y;\phi) + C(1/\phi;\phi)\mathrm{RER} = \left[\langle gf/\phi\rangle\int_\Sigma Pf\,\mathrm{d}A - \langle f^2/\phi\rangle\int_\Sigma Pg\,\mathrm{d}A\right], \tag{S9}$$

noting that f, g depend on $\mathbf{d}$ via (S1).

Returning to the general expressions (S4), (S6), we see that extension is regulated by $\langle\phi^{-1}\rangle$ and $\langle Y\rangle$, while bending is regulated by $\langle fY\rangle$, $\langle f/\phi\rangle$, $\langle f^2/\phi\rangle$ and $\int_\Sigma Pf\,\mathrm{d}A$. We can use these expressions to determine the relative contributions of different cell layers to generation of bending moment, for any given root geometry.

Henceforth we will assume for simplicity that P is uniform across the root, so that $\overline{P} = P$ and $\int_\Sigma Pf\mathrm{d}A = P\int_\Sigma f\mathrm{d}A$. We now address some special cases.

S1.2 Small transverse gradients in extensibility and yield

If there are only small variations in ϕ and Y across the root, then we can write $\phi = \overline{\phi} + \hat{\phi}$, $Y = \overline{Y} + \hat{Y}$ where $\langle\hat{\phi}\rangle = 0$, $\langle\hat{Y}\rangle = 0$ and

$$\overline{\phi} = \langle\phi\rangle/\mathcal{L}, \quad \overline{Y} = \langle Y\rangle/\mathcal{L},$$

where, recall, $\mathcal{L} = \int_N \mathrm{d}s$. We also note that $\langle f\phi\rangle = \langle f\hat{\phi}\rangle$, $\langle f/\phi\rangle \approx -\langle f\hat{\phi}\rangle/\overline{\phi}^2$, $\langle fY\rangle = \langle f\hat{Y}\rangle$, $\langle g\phi\rangle = \langle g\hat{\phi}\rangle$ and $\langle gY\rangle = \langle g\hat{Y}\rangle$. Then neglecting quantities that are quadratic in κf, $\hat{\phi}$ and $\hat{Y}$, (S4) becomes

$$\mathrm{RER} = \overline{\phi}\left(\frac{P\mathcal{A}}{\mathcal{L}} - \overline{Y}\right) \qquad (P\mathcal{A} > \overline{Y}\mathcal{L}). \tag{S10}$$

From (S8), we see that $C(Y;\phi) \approx C(\hat{Y};\overline{\phi}) \approx C(\hat{Y};1)/\overline{\phi}$ and $C(1/\phi;\phi) \approx C(1/\phi;1)/\overline{\phi} \approx -C(\hat{\phi};1)/\overline{\phi}^3$, so that (S9) becomes

$$C(\hat{Y};1) - \frac{C(\hat{\phi};1)}{\overline{\phi}}\left(\frac{P\mathcal{A}}{\mathcal{L}} - \overline{Y}\right) = P\left[\langle gf\rangle \int_\Sigma f\,\mathrm{d}A - \langle f^2\rangle \int_\Sigma g\,\mathrm{d}A\right]. \tag{S11}$$

In this case, from (S6) we see that bending evolves according to

$$\mathrm{CGR} = \frac{\langle f\phi\rangle}{\langle f^2\rangle}\left(\frac{P\mathcal{A}}{\mathcal{L}} - \overline{Y}\right) - \overline{\phi}\frac{\langle fY\rangle}{\langle f^2\rangle} + \overline{\phi}\frac{P}{\langle f^2\rangle}\int_\Sigma f\,\mathrm{d}A. \tag{S12}$$

As anticipated, curvature is generated by the first moments of extensibility and yield. Note that if ϕ and Y are uniform, the final term in (S12) shows how geometric nonuniformities can also generate bending.

If, in addition, N is symmetric, then $\int_\Sigma f\,\mathrm{d}A = 0$, $\int_\Sigma g\,\mathrm{d}A = 0$ and $\langle gf\rangle = 0$, so that (S11) reduces to

$$-\langle gY\rangle + \langle g\phi\rangle\frac{1}{\overline{\phi}}\left(\frac{P\mathcal{A}}{\mathcal{L}} - \overline{Y}\right) = 0 \qquad (P\mathcal{A} > \overline{Y}\mathcal{L}) \tag{S13}$$

upon substituting $C(Y;1)$, $C(\phi,1)$ from (S8). This shows how gradients in yield and extensibility together contribute to determining the direction of bending. For example, if $\hat{Y}$ is proportional to $\hat{\phi}$, then (S13) demands that $\langle gY\rangle = 0$, $\langle g\phi\rangle = 0$, conditions that determine $\mathbf{d}$.

Notes S2: Idealised Root Geometry

We now use the framework described in Notes S1 and consider an idealised root geometry to investigate the effect different cell files have on the composite behaviour of the root (Fig. S2). We take an axial cross-section and dissect it into 8 identical sectors (labelled $A - H$ anticlockwise in Fig. S2) each containing three epidermal cells, one cortical cell, one endodermal cell, two pericycle cells and one stele cell. In this representation, there are fewer cells in the vasculature than can be seen in Fig 2(b); however, the vascular cells are not thought to make a significant contribution to the mechanical properties of the root. We assume sufficient

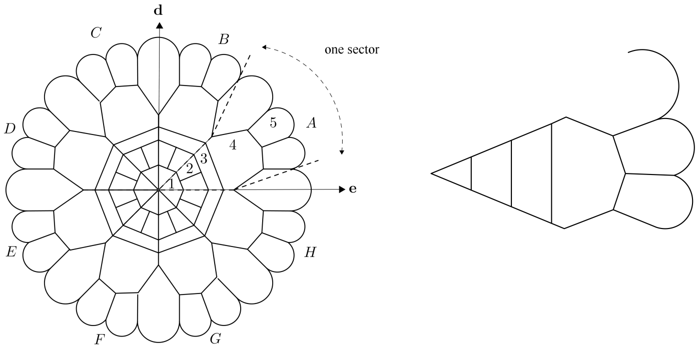

(a) (b)

Figure S2: (a) The polyhedral array of cells in an idealised root cross-section and (b) a "slice" of root which combines to make an entire disk. Labels $A - H$ denote the location of a "slice" of root in the cross section, whilst $1 - 5$ label the cell type from inner to outer, with 1 denoting the stele and 5 denoting the epidermis.

symmetry that this cross-section can be extended to describe the root structure along the entire elongation zone. The turgor pressure, P, throughout the cells is assumed uniform, so only the outer wall of the epidermal layer will be curved; the other (internal) walls will be flat. We are concerned here only with out-of-plane tensions, but note that in-plane tensions must be such as to balance when they act at each vertex.

We describe a slice of the cross-section using cylindrical polar coordinates r, θ, z. The following geometric variables are shown in Fig. S3. We label each cell type such that cell type $i = 1$ is the inner (stele) cell file, and cell type $i = 5$ is the outer (epidermal) layer. We label the azimuthal walls on the outer edge of cell i with subscript zr and superscript (i) and the radial cell walls of cell i with subscript $z\theta$ and superscript (i). Thus in Fig. S3 we denote the thickness of azimuthal walls on the outer edge of cell i as $h_{zr}^{(i)}$ and the thickness of radial cell walls of cell i as $h_{z\theta}^{(i)}$. We denote the two epidermal cells which lie over a single cortical cell with subscript OUT and that which lies over the junction of two cortical cells with subscript IN. Radii $r = R^{(i)}$, $i = 1, \dots 8$ denote cell wall junctions: for notational convenience we define $R^{(0)} = 0$. We denote the radius of curvature of the outer walls by ρ_{IN}, ρ_{OUT} such that the arc of the wall subtends an angle β_{IN}, β_{OUT}, whilst γ denotes the angle that the junction between "inner" and "outer" epidermal cells subtend at the axis of symmetry of the segment. The lengths of the side and cross walls are denoted $l^{(i)}$, $i = 1 \dots 4$ and $w^{(i)}$, $i = 1 \dots 3$ respectively, with $l_{IN}^{(2)}$ denoting the

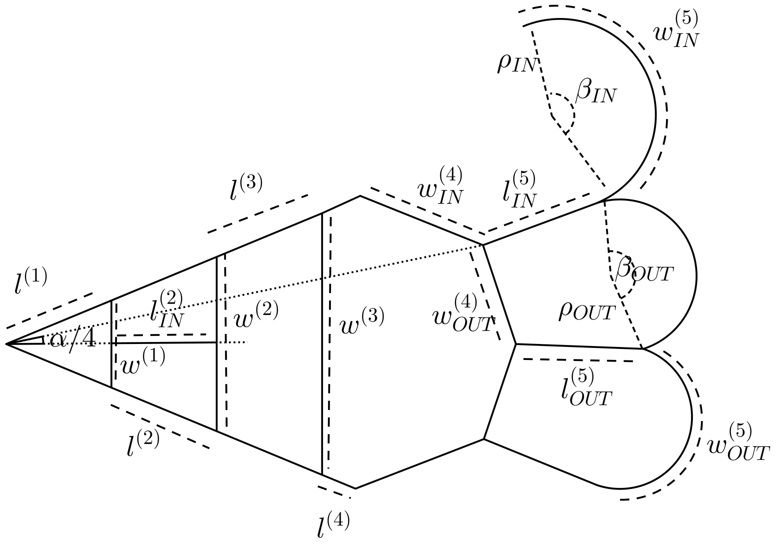

(a)

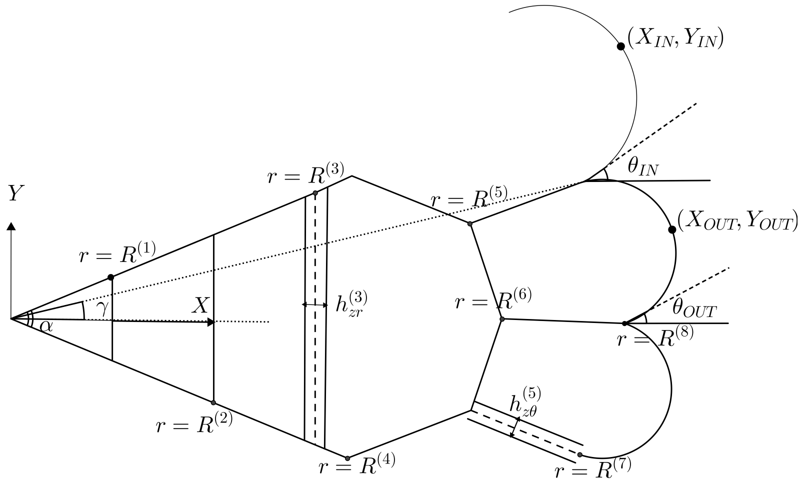

(b)

Figure S3: Two ‘slices’ of a root cross-section with geometric labels.

length of the cortical side wall internal to the sector, $l_{IN}^{(5)}$, $l_{OUT}^{(5)}$ denoting the length of the radial cross walls of the respective epidermal cells and $w_{IN}^{(4)}$, $w_{OUT}^{(4)}$, $w_{IN}^{(5)}$, $w_{OUT}^{(5)}$ denoting the length of the internal and external cross walls of the respective epidermal cells.

Using geometric relationships, we can express all lengths and angles in terms of the radial wall positions $R^{(i)}$, the outer wall lengths $w_{IN}^{(5)}$, $w_{OUT}^{(5)}$, and the angle γ according to

$$
\begin{aligned}
l^{(i)} &= R^{(i)} - R^{(i-1)} \quad \text{for} \quad i = 1 \ldots 4, && \text{(S14)}\\
w^{(i)} &= R^{(i)} \frac{\sin\alpha}{\cos(\alpha/2)} \quad \text{for} \quad i = 1 \ldots 3, && \text{(S15)}\\
l_{IN}^{(2)} &= \left(R^{(1)} - R^{(2)}\right)\cos(\alpha/2), && \text{(S16)}\\
l_{IN}^{(5)} &= \sqrt{R^{(6)^2} + R^{(5)^2} - 2R^{(6)}R^{(5)}\cos(\gamma - \alpha/4)}, && \text{(S17)}\\
l_{OUT}^{(5)} &= R^{(8)} - R^{(6)}, && \text{(S18)}\\
w_{OUT}^{(4)} &= \sqrt{R^{(5)^2} + R^{(6)^2} - R^{(5)}R^{(6)}\cos(\alpha/2)}, && \text{(S19)}\\
w_{IN}^{(4)} &= \sqrt{R^{(4)^2} + R^{(5)^2} - R^{(4)}R^{(5)}\cos(\alpha/2)}, && \text{(S20)}\\
w_{OUT}^{(5)} &= \beta_{OUT}\rho_{OUT}, && \text{(S21)}\\
w_{IN}^{(5)} &= \beta_{IN}\rho_{IN}; && \text{(S22)}
\end{aligned}
$$

see Fig. S3. We currently have only two equations (S21), (S22) for the four unknowns ρ_{OUT}, ρ_{IN}, β_{OUT}, β_{IN}, and thus final relationships must be specified to determine them. These are given by the requirement that the outer walls are arcs of circles of a specified length passing through specified points. This equates to the conditions

$$
\begin{aligned}
R^{(7)}\cos\gamma &= R^{(8)} + \rho_{OUT}\left(\sin(\beta_{OUT} + \theta_{OUT}) - \sin\theta_{OUT}\right), && \text{(S23)}\\
R^{(7)}\sin\gamma &= \rho_{OUT}\left(\cos\theta_{OUT} - \cos(\beta_{OUT} + \theta_{OUT})\right), && \text{(S24)}\\
R^{(7)}\cos(\alpha - \gamma) &= R^{(7)}\cos\gamma + \rho_{IN}\left(\sin(\beta_{IN} + \theta_{IN}) - \sin\theta_{IN}\right), && \text{(S25)}\\
R^{(7)}\sin(\alpha - \gamma) &= R^{(7)}\sin\gamma + \rho_{IN}\left(\cos\theta_{IN} - \cos(\beta_{IN} + \theta_{IN})\right), && \text{(S26)}
\end{aligned}
$$

where θ_{OUT} and θ_{IN} are the angles made between the axis of symmetry of the slice and the outer epidermal cell wall as defined in Fig. S3. The area of the slice can then be found as

$$
\begin{aligned}
\frac{\mathcal{A}}{8} = {} & R^{(8)}R^{(7)}\sin\gamma + \rho_{OUT}^2\left(\beta_{OUT} - \sin\beta_{OUT}\right)\\
& + \frac{R^{(7)^2}}{2}\sin(\alpha - 2\gamma) + \frac{\rho_{IN}^2}{2}\left(\beta_{IN} - \sin\beta_{IN}\right). \qquad \text{(S27)}
\end{aligned}
$$

We undertake a simple check of these complex idealised geometry calculations by approximating azimuthal cell walls as a series of circular rings of cell wall material and radial walls as true radii. Upon calculating the total length of cell wall ($\mathcal{L}$) and the total cross sectional area of the root ($\mathcal{A}$) using both the complex and simple geometries we find that the two methods differ by under 8% (length) and 5% (area), giving confidence in our calculations.

We parameterise the outer epidermal walls in the segment (in $X-Y$ coordinates where $(0,0)$ is located at the centre of the root cross-section) via

$$X_{OUT} = R^{(8)} + \rho_{OUT}\left(\sin\left(\eta/\rho_{OUT} + \theta_{OUT}\right) - \sin\theta_{OUT}\right), \tag{S28}$$

$$Y_{OUT} = \rho_{OUT}\left(\cos\theta_{OUT} - \cos\left(\eta/\rho_{OUT} + \theta_{OUT}\right)\right), \tag{S29}$$

$$X_{IN} = R^{(7)}\cos\gamma + \rho_{IN}\left(\sin\left(\nu/\rho_{IN} + \theta_{IN}\right) - \sin\theta_{IN}\right), \tag{S30}$$

$$Y_{IN} = R^{(7)}\sin\gamma + \rho_{IN}\left(\cos\theta_{IN} - \cos\left(\nu/\rho_{IN} + \theta_{IN}\right)\right), \tag{S31}$$

where $\eta = 0 \dots w_{OUT}^{(5)}$ and $\nu = 0 \dots w_{IN}^{(5)}$.

We want to investigate the effects on extension and bending of changing the mechanical properties of individual cells. Since the domain N is symmetric, $\mathbf{x}_0$ (the centroid of Σ) lies at $(0,0)$. We prescribe the viscosity and yield stress of the walls of each individual cell ($\mu^{(i)}$, $y^{(i)}$). These properties consist of a common component for each cell in cell file i ($\bar{\mu}^{(i)}$, $\bar{y}^{(i)}$, which govern elongation) combined with variations which average to zero across the cross section ($\hat{\mu}^{(i)}$, $\hat{y}^{(i)}$, which govern bending). We assume that the middle lamella between two neighbouring cells lies in the centre of the composite cell wall so that the mechanical properties of each cell contribute equally to the composite properties.

Recalling that $Y(\mathbf{x}) = \int_0^{h(\mathbf{x})} y \;\mathrm{d}n$ and $\phi(\mathbf{x}) = 1/\int_0^{h(\mathbf{x})} 4\mu(n)\;\mathrm{d}n$ (Dyson & Jensen 2010), the base extensibility and yield of each individual cell wall segment in a given cell file is therefore

$$\phi_{zr}^{(i)} = \frac{1}{2\left(\bar{\mu}^{(i)} + \bar{\mu}^{(i+1)}\right)h_{zr}^{(i)}} \quad i = 1\dots 4, \tag{S32}$$

$$\phi_{zr}^{(5)} = \frac{1}{4\bar{\mu}^{(5)}h_{zr}^{(5)}}, \tag{S33}$$

$$\phi_{z\theta}^{(i)} = \frac{1}{4\bar{\mu}^{(i)}h_{z\theta}^{(i)}} \quad i = 1\dots 5, \tag{S34}$$

$$Y_{zr}^{(i)} = \frac{\left(\bar{y}_{zr}^{(i)} + \bar{y}_{zr}^{(i+1)}\right)h_{zr}^{(i)}}{2} \quad i = 1\dots 4, \tag{S35}$$

$$Y_{zr}^{(5)} = \bar{y}_{zr}^{(5)}h_{zr}^{(5)}, \tag{S36}$$

$$Y_{z\theta}^{(i)} = \bar{y}_{z\theta}^{(i)}h_{z\theta}^{(i)} \quad i = 1\dots 5, \tag{S37}$$

using the same naming convention as for the wall thicknesses.

From (S4) we then find the effective extensibility and yield to be

$$\begin{aligned}\frac{1}{\phi_{\text{eff}}} &= \sum_{i=1}^{3}\frac{w^{(i)}}{\phi_{zr}^{(i)}}+\sum_{i=1}^{4}\frac{l^{(i)}}{\phi_{z\theta}^{(i)}}+\frac{l_{INT}^{(2)}}{\phi_{z\theta}^{(2)}}+\frac{2l_{IN}^{(5)}+l_{OUT}^{(5)}}{\phi_{z\theta}^{(5)}}+\frac{2\left(w_{IN}^{(4)}+w_{OUT}^{(4)}\right)}{\phi_{zr}^{(4)}}\\ &\quad+\frac{\left(w_{IN}^{5}+2w_{OUT}^{(5)}\right)}{\phi_{zr}^{(5)}}, \qquad \text{(S38)}\\ Y_{\text{eff}} &= \sum_{i=1}^{3}Y_{zr}^{(i)}w^{(i)}+\sum_{i=1}^{4}Y_{z\theta}^{(i)}l^{(i)}+Y_{z\theta}^{(2)}l_{INT}^{(2)}+(2l_{IN}^{(5)}+l_{OUT}^{(5)})Y_{z\theta}^{(5)}\\ &\quad+2Y_{zr}^{(4)}\left(w_{IN}^{(4)}+w_{OUT}^{(4)}\right)+Y_{zr}^{(5)}\left(w_{IN}^{5}+2w_{OUT}^{(5)}\right). \qquad \text{(S39)}\end{aligned}$$

Due to the reciprocals present in (S38), the extensibility is dominated by the minimum extensibility of the system; if one extensibility is significantly smaller than the rest, this will be the dominant term. In contrast, the effective yield stress (S39) is given by a linear sum of the yield stresses; if one yield stress is significantly higher this will be the dominant term.

To investigate bending we assume that the variations to the base material properties of cell i ($\hat{\mu}^{(i)}$, $\hat{Y}^{(i)}$) are distributed symmetrically across the root; *i.e.* we take identical perturbations to material properties when reflected in one axis of symmetry (taken, without loss of generality, to be the line between segments H and A in Fig S2a) whilst on either side of a line perpendicular to this axis enhanced material properties on one side are exactly compensated for by reduced material properties on the other. This defines the axes $\mathbf{e}$ and $\mathbf{d}$ respectively, and is equivalent to the statement

$$\hat{\mu}(f,g) = -\hat{\mu}(-f,g) = \hat{\mu}(f,-g), \qquad \text{(S40)}$$
$$\hat{Y}(f,g) = -\hat{Y}(-f,g) = \hat{Y}(f,-g) \qquad \text{(S41)}$$

where $\mathbf{x} = f\mathbf{e} + g\mathbf{d}$ and $\hat{\mu}$, $\hat{Y}$ denote the distribution of properties $\hat{\mu}^{(i)}$, $\hat{Y}^{(i)}$. It is easily seen that these definitions satisfy the conditions $\langle\hat{\mu}\rangle = 0$, $\langle\hat{Y}\rangle = 0$.

With the symmetries in the profile of material properties as described above, the bending of the root can be determined by considering the upper right quadrant only since all other terms are either identical or the negative of those already found. Assuming this distribution of mechanical properties, (S7) is identically satisfied and (S6) determines the development of curvature in the segment. There are no geometric nonlinearities, so the RHS of (S7) is zero, and thus we may write

$$\frac{-\langle f^2/\phi\rangle}{\text{RER}}\text{CGR} = \langle f/\phi\rangle, \qquad \text{(S42)}$$

where we neglect any variations in yield, or

$$-\langle f^2/\phi\rangle \text{CGR} \;=\; \langle fY\rangle, \tag{S43}$$

where we neglect any variations in viscosity. In both (S42), (S43) only the RHS changes with variations across a cross section, and it is these terms which drive the changes in curvature. The calculation of these terms is simple but lengthy given the parameterisation (S28-S31) so we neglect the details for brevity, merely showing the graph Fig. 4c.

Notes S3: Bending induced by asymmetric extensibility

To illustrate how growth and curvature generation interact in Eq. (4) of the main text to produce the total bending angle $\Delta\theta$ (see Fig. S1), we write arc-length along the root midline as $s = s_0 + s_1 S$, where $s = s_0$ defines the start of the EZ, and s_1 gives the length of the EZ. It is also convenient to write time as $t = (s_1 c/l_0)T$, where T is dimensionless. We assume cells enter the EZ at length l_0 and exit at length βl_0 (where $\beta \approx 30$ for *Arabidopsis*). We write

$$c\text{RER} = (l_0/s_1)G(S) \quad \text{for} \quad 0 \le S \le 1, \tag{S44}$$

the pattern of growth across the EZ but we do not give a specific form in what follows, except to impose the conditions $G(0) = 0$, $G(1) = 0$ and $G > 0$ within the EZ. Here we adopt a simple functional definition of the EZ as the region wherein cell expansion takes place. Writing $l = l_0 L(S)$, (4a) becomes

$$\frac{\mathrm{d}L}{\mathrm{d}S} = G(S) \tag{S45}$$

implying

$$L(S) = 1 + \int_0^S G(z)\,\mathrm{d}z \tag{S46}$$

with

$$L(1) \equiv \beta = 1 + \int_0^1 G(z)\,\mathrm{d}z. \tag{S47}$$

Thus G defines a steady distribution of cell lengths, and equivalently prescribes the speed at which cells move through the EZ.

We assume that curvature is generated by transverse gradients in ϕ alone, and write $-\langle f/\phi\rangle/\langle f^2/\phi\rangle = A(S,T)$ (see (S6)), where A has dimensions of inverse distance. Writing CGR $= A\,$RER, we can re-express (5) as

$$\frac{\mathrm{d}\kappa}{\mathrm{d}T} = A(S,T)\,G(S) \quad \text{on} \quad \frac{\mathrm{d}S}{\mathrm{d}T} = L. \tag{S48}$$

Here we are envisaging a root that is undergoing steady elongation, represented by $G(S)$, during which it undergoes unsteady tropic bending, controlled by transient asymmetries in extensibility represented by $A(S,T)$. To illustrate, for a root with a symmetric cross section, for which $\langle f\rangle = \langle f^3\rangle = 0$, a candidate distribution of extensibility that is consistent with this picture has the form

$$\phi(S,T) \propto \frac{G(S)}{1 - A(S,T)f}. \tag{S49}$$

We are assuming uniform turgor and yield force, and require $|Af| < 1$. Eq. (S49) illustrates how unsteady asymmetries that drive bending modulate the steady average extensibility that drives elongation.

We define the characteristics $S(T;T_0)$ of (S48) satisfying $S(T_0;T_0) = 0$ by

$$\frac{\mathrm{d}S}{\mathrm{d}T} = \begin{cases} 1 & (S < 0) \\ 1 + \int_0^S G(z)\,\mathrm{d}z & (0 < S < 1) \\ \beta & (S > 1). \end{cases} \tag{S50}$$

T_0 gives the time at which the characteristic enters the EZ and is used to parameterize characteristics. Since (S50) is independent of T_0, we can express characteristics in terms of a family of monotonic functions $S(T;T_0) = F(T - T_0)$, where

$$F(\hat{T}) = \hat{T} \qquad (\hat{T} < 0) \tag{S51}$$

$$\int_0^F \frac{\mathrm{d}\mathcal{F}}{1 + \int_0^{\mathcal{F}} G(z)\mathrm{d}z} = \hat{T} \qquad (0 < \hat{T} < T_{\mathrm{t}}) \tag{S52}$$

$$F(\hat{T}) = 1 + \beta(\hat{T} - T_{\mathrm{t}}) \qquad (\hat{T} > T_{\mathrm{t}}), \tag{S53}$$

where $\hat{T} \equiv T - T_0$. The dimensionless transit time through the EZ is

$$T_{\mathrm{t}} = \int_0^1 \frac{\mathrm{d}\mathcal{F}}{1 + \int_0^{\mathcal{F}} G(z)\,\mathrm{d}z}. \tag{S54}$$

Because $1 < L < \beta$ in $0 < S < 1$, we can write $T_{\mathrm{t}} = \gamma/\beta$ for some factor $\gamma > 1$, making the dimensional transit time

$$t_0 = \frac{s_1}{\beta l_0}\gamma c. \tag{S55}$$

The function F is illustrated in Fig. S4, showing dimensionless cell length (or equivalently dimensionless cell transit speed) as a function of time through the

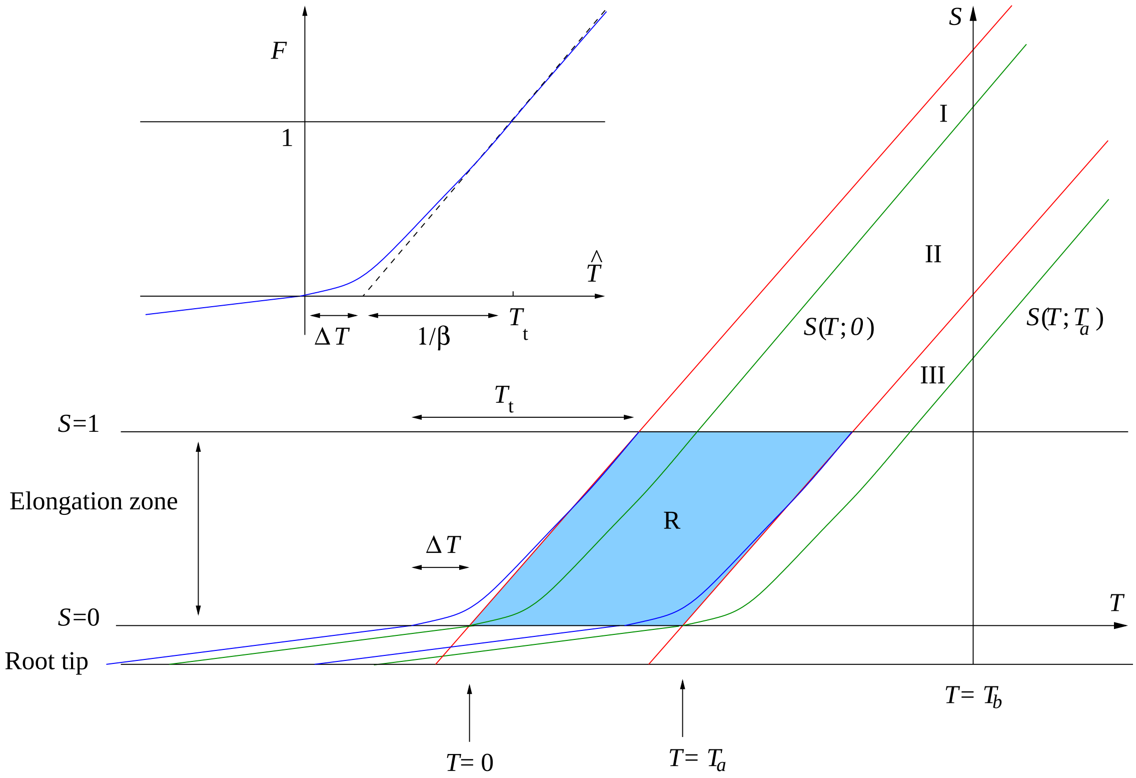

Figure S4: A space-time diagram, plotting distance S along the root (scaled on the length of the EZ) against time T (scaled against the elongation timescale s_1c/l_0). Cells move through the EZ along characteristic curves. When growth is quasi-steady, these have a universal form (as shown by the function $F(\hat{T})$ in the inset, which plots location along the root F versus time since entering the EZ $\hat{T}$; T_t is the transit time through the EZ). In the (T, S) graph, the EZ occupies $0 < S < 1$; the slope of characteristics illustrates the speed of cells relative to the root tip (increasing across the EZ from 1 to β, when scaled against l_0/c); the distance between adjacent characteristics illustrates the corresponding elongation of cells as they pass through the EZ (from length 1 to β, when scaled against l_0). We consider a curvature-inducing signal that initiates at $S = 0$ for time $0 < T < T_a$. This either moves steadily along the root (confined by straight red lines, which intersects the EZ in region R), or is swept along by cells as they transit the EZ (between green characteristics $S(T; 0)$ and $S(T; T_a)$). In either case, the root curvature at some later time T_b is determined by tracking the history of cells that have passed through the EZ, lying in regions I, II and III.

EZ. Note that for $0 < F < 1$, $F'(\hat{T}) = 1 + \int_0^F G(z)\,\mathrm{d}z$ and $F''(\hat{T}) = G(F)F'(\hat{T})$ implying that

$$\int_0^1 G(z)\,\mathrm{d}z = \int_0^1 \frac{F''(\hat{T})}{F'(\hat{T})}\,\mathrm{d}F = \int_0^{T_\mathrm{t}} F''(\hat{T})\,\mathrm{d}\hat{T} = \left[F'(\hat{T})\right]_0^{T_\mathrm{t}} = \beta - 1 \qquad \text{(S56)}$$

as required from (S47). Note also that extrapolating the large-$\hat{T}$ form of F back to $F = 0$ gives an intersection at $\hat{T} = \Delta T \equiv T_\mathrm{t} - (1/\beta) = (\gamma - 1)/\beta$. This corresponds to the difference between the actual transit time across the EZ and the transit time of fully elongated cells across the same distance.

To simulate a gravitropic motion, we assume a wave-like signal that passes shootwards as a result of a transient gravity stimulus at the root tip. Thus the candidate exensibility (S49) maintains its steady mean profile $G(S)$ but we suppose a localised asymmetric perturbation A travels shootwards. For simplicity, we assume that the perturbation travels with uniform dimensional wave-speed $\beta l_0/c$ relative to the root tip, which matches the speed at which expanded cells leave the EZ; this assumption is revisited below. Thus we write A in (S48, S49) as $A(\omega)$ where $\omega \equiv T - (S/\beta)$. Then on the characteristic labelled by T_0, the curvature distribution $\kappa(S, T)$ satisfies (from (S6))

$$\frac{\mathrm{d}\kappa}{\mathrm{d}T} = A(\omega)G(S) \quad \text{on} \quad S(T; T_0) = F(\hat{T}) \quad \text{where} \quad \hat{T} \equiv T - T_0. \qquad \text{(S57)}$$

Thus

$$\kappa = \int A\left(T - \frac{S}{\beta}\right) G(S)\,\mathrm{d}T, \qquad \text{(S58)}$$

where the integral must be evaluated along the relevant characteristic.

We now demonstrate explicitly how a bending angle is generated for the simple case in which the wave A is a top-hat function satisfying

$$A(\omega) = \begin{cases} A_0 & 0 < \omega < T_a, \\ 0 & \text{otherwise.} \end{cases} \qquad \text{(S59)}$$

Here T_a is related to the dimensional time of action of the signal via $t_a = s_1 c T_a / l_0$. The corresponding candidate extensibility distribution (S49) maintains a smooth average profile $G(S)$, but we now assume the asymmetric perturbation that travels shootwards is an isolated pulse, within which ϕ is uniformly elevated (reduced) on the outer (inner) walls of the root. Since the ultimate bending angle depends on a spatial integral of the curvature, and the curvature is determined by a further integral (S58) over the pulse, we anticipate that more refined details of the shape of the perturbation will not be significant.

In the (T, S)-plane (Fig. S4), we identify two strips. The EZ lies in $0 < S < 1$; here the RER is non-zero, allowing cells to elongate. In the transverse strip $\beta(T - T_a) < S < \beta T$ is the wave of the curvature-inducing signal (S59). Where the strips intersect, then curvature is generated. We denote the intersection region as R.

We distinguish three classes of characteristics: those that enter R in $0 < S < 1$ and leave it along $S = 1$ (I); those that enter R along $S = 0$ and leave it along $S = 1$ (II); and those that enter R along $S = 0$ and leave it in $0 < S < 1$. From (S58), the curvature on exiting region I is (noting that for fixed T_0, $\mathrm{d}T = \mathrm{d}\hat{T}$)

$$\kappa_{\mathrm{I}} = A_0 \int_{\hat{T}_{\mathrm{in}}}^{T_{\mathrm{t}}} G \,\mathrm{d}\hat{T}. \tag{S60}$$

This integral is parameterized by $0 < \hat{T}_{\mathrm{in}} < 1$, the $\hat{T}$ value at which a characteristic enters R along $S = \beta T$, which satisfies

$$F(\hat{T}_{\mathrm{in}}) = \beta(\hat{T}_{\mathrm{in}} + T_0); \tag{S61}$$

the characteristic leaves R at $T = T_0 + T_{\mathrm{t}}$. The range of T_0 is

$$-\Delta T < T_0 < 0. \tag{S62}$$

The curvature on exiting region II is uniform, satisfying on a given characteristic

$$\kappa_{\mathrm{II}} = A_0 \int_0^{T_{\mathrm{t}}} G \,\mathrm{d}T = A_0 \int_0^1 \frac{G}{L} \,\mathrm{d}S$$
$$= A_0 \int_0^1 \frac{\mathrm{d}L/\mathrm{d}S}{L} \,\mathrm{d}S = A_0 \int_0^{\beta} \frac{\mathrm{d}L}{L} = A_0 \log \beta. \tag{S63}$$

The curvature on exiting region III is

$$\kappa_{\mathrm{III}} = A_0 \int_0^{\hat{T}_{\mathrm{out}}} G \,\mathrm{d}\hat{T}, \tag{S64}$$

parameterized by $0 < \hat{T}_{\mathrm{out}} < 1$, where $\hat{T}_{\mathrm{out}}$ is the $\hat{T}$ value at which the characteristic passing through $S = 0$, $T = T_0$ leaves R on $z = \beta(T - T_a)$, i.e.

$$F(\hat{T}_{\mathrm{out}}) = \beta(\hat{T}_{\mathrm{out}} + T_0 - T_a), \tag{S65}$$

for T_0 in the range $T_a - \Delta T < T_0 < T_a$.

We then evaluate the shape at a large time T, by assessing the curvature distribution along a line $T =$ constant in the (S, T)-plane. The curvature is zero

everywhere except where it intersects the characteristics emerging from regions I–III. The total bending angle (see Fig. S1) is then

$$\Delta\theta = \int \kappa\,\mathrm{d}s = s_1\left(\int_{III} + \int_{II} + \int_{I}\right)\kappa\,\mathrm{d}S. \tag{S66}$$

We re-parameterize each integral using T for II, $\hat{T}_{\mathrm{in}}$ for I and $\hat{T}_{\mathrm{out}}$ for III.

In region II, the curvature is uniform along

$$\beta(T - T_a) < S < \beta(T - \Delta T). \tag{S67}$$

Tracking back along characteristics, we set $\mathrm{d}S = \beta \mathrm{d}T$ and integrate along $S = 1$ over a distance $T_a - \Delta T$. Thus, from (S63)

$$\Delta\theta_{\mathrm{II}} = A_0 s_1 \beta \log \beta (T_a - \Delta T) \tag{S68}$$

Here we are assuming that T_a is large enough for regions I and III to be distinct.

In region I, parameterising the integral in (S66) using $\hat{T}_{in}$ via (S61) we obtain

$$\Delta\theta_{\mathrm{I}} = A_0 s_1 \beta \int_{-\Delta T}^{0} \mathrm{d}T_0 \int_{\hat{T}_{\mathrm{in}}}^{T_{\mathrm{t}}} G(\hat{T}')\,\mathrm{d}\hat{T}' = A_0 s_1 \int_0^{T_{\mathrm{t}}} \mathrm{d}\hat{T}_{\mathrm{in}}\,(\beta - F') \int_{\hat{T}_{\mathrm{in}}}^{T_{\mathrm{t}}} G(\hat{T}')\,\mathrm{d}\hat{T}', \tag{S69}$$

while in region III, analogously,

$$\Delta\theta_{\mathrm{III}} = A_0 s_1 \beta \int_{T_a - \Delta T}^{T_a} \mathrm{d}T_0 \int_0^{\hat{T}_{\mathrm{out}}} G\,\mathrm{d}\hat{T} = A_0 s_1 \int_0^{T_{\mathrm{t}}} \mathrm{d}\hat{T}_{\mathrm{out}}\,(\beta - F') \int_0^{\hat{T}_{\mathrm{out}}} G(\hat{T}')\,\mathrm{d}\hat{T}'. \tag{S70}$$

These contributions can be added to give

$$\begin{aligned}
\Delta\theta &= A_0 s_1 \left[\beta \log \beta (T_a - \Delta T) + \int_0^{T_{\mathrm{t}}} \mathrm{d}\hat{T}\,(\beta - F') \int_0^{T_{\mathrm{t}}} G(\hat{T}')\,\mathrm{d}\hat{T}'\right] && \text{(S71)}\\
&= A_0 s_1 \left[\beta \log \beta (T_a - \Delta T) + \int_0^{T_{\mathrm{t}}} \mathrm{d}\hat{T}\,(\beta - F') \int_0^{1} \frac{\mathrm{d}L/\mathrm{d}S}{L}\,\mathrm{d}S\right] && \text{(S72)}\\
&= A_0 s_1 \log \beta \left[\beta (T_a - \Delta T) + \int_0^{T_{\mathrm{t}}} \mathrm{d}\hat{T}\,(\beta - F')\right] && \text{(S73)}\\
&= A_0 s_1 \log \beta \left[\beta (T_a - \Delta T) + \beta T_{\mathrm{t}} - 1\right] = A_0 s_1 T_a \beta \log \beta. && \text{(S74)}
\end{aligned}$$

Expressed in terms of the dimensional time of activation of the wave, $t_a = s_1 c T_a / l_0$, the total bending angle becomes

$$\Delta\theta = \frac{A_0 l_0}{c} t_a \beta \log \beta, \tag{S75}$$

corresponding to Eq. (7) of the main text.

An alternative, and even simpler, assumption is that the gravitropic stimulus at the root tip is switched on at $T = 0$, off at $T = T_a$, and is applied to cells entering the EZ during that interval, without being transported from cell to cell. Thus the asymmetric perturbation $A(S, T)$ in (S49) again travels as a localised pulse, but now at the same speed as the cells moving through the EZ. The net bending angle again satisfies (S75), as in this case all softened cells leaving the EZ have curvature $A_0 \log \beta$ and they occupy a length of root equal to $\beta l_0 t_a / c$. Remarkably, therefore, the net bending angle is insensitive to the speed with which the localised signal moves along the root[1]. We can use this example to consider how the angle rises to this final state. Consider in particular the characteristics $T_0 = 0$ and $T_0 = T_a$, which bound the softened region (shown as green in Fig. S4). Between these characteristics, and in the EZ,

$$\kappa = A_0 \log L|_{T_0} \quad (0 < T_0 < T_a). \tag{S76}$$

Thus the curvature is greatest along the characteristic $T_0 = 0$. The net bending angle is obtained by integrating this curvature with respect to Σ for fixed T between these characteristics. Noting that $\mathrm{d}S/\mathrm{d}T = L$ for fixed T_0, it follows that

$$\frac{\mathrm{d}\theta}{\mathrm{d}T} = A_0 s_1 \frac{\mathrm{d}}{\mathrm{d}T} \int_{F(T-T_a)}^{F(T)} \log L \, \mathrm{d}S = A_0 s_1 \left[L \log L \right]_{T_0=T_a}^{T_0=0}. \tag{S77}$$

Thus the angle reaches its maximum $\Delta\theta$ once both characteristics have left the EZ.

We can identify the angle generated during the transit time T_t as

$$\theta_0 = A_0 s_1 \int_0^1 \log L \, \mathrm{d}S. \tag{S78}$$

Since $1 < L < \beta$ in the EZ, we can write (S78) as $\theta_0 = A_0 s_1 \mathcal{G} \log \beta$ for some factor $0 < \mathcal{G} < 1$ that depends on the precise form of $G(S)$. Then from (S55) we can write

$$\theta_0 = A_0 l_0 \frac{t_0}{c} \frac{\mathcal{G}}{\gamma} \beta \log \beta = A_0 s_1 \mathcal{G} \log \beta. \tag{S79}$$

This is shorter than the time predicted by (S75) with $t_a = t_0$ by a factor $\mathcal{G}/\gamma < 1$. It represents the angle turned by the root once the initial softening signal has first crossed the EZ. Likewise, if $t_a > t_0$, θ_0 represents the angle turned by the root after then signal for softening switches off at $t = t_a$.

[1]The result (S75) can also be derived for a stimulus that is stationary with respect to the root tip, provided its duration exceeds the transit time through the EZ ($T_a > T_t$).

Notes S4: Pressure probe data

The data plotted in Fig. 3a is given in Table S1.

Notes S5: Further cell wall thickness analysis

Comparing the cell wall thickness data between the early meristematic zone (zone 1A) and the late meristematic zone (zone 1B), the general pattern with only a few exceptions is that the cell walls in zone 1A are significantly thinner than the corresponding cell walls in zone 1B. We note that in zone 1A, at the junctions between the cortex and its neighbouring cell files, there is a statistically significant difference between a) the thickness of the wall between the cortex and the middle lamella, and b) the thickness of the wall between the middle lamella and the endodermis/epidermis. At both the endodermis/cortex and cortex/epidermis junctions, the cell walls are thicker on the side of the cortex. Expressing the difference as a percentage change in the mean cell thickness, we find that in zone 1A the walls between the cortex and the endodermis are 17.5 % thicker on the side of the cortex than on the side of the endodermis, and the walls between the cortex and the epidermis are 11.7 % thicker on the side of the cortex than on the side of the epidermis. Since the differences in cell wall thickness for the walls on either side of the cortex are not statistically significant in zone 1B, we pool the results for these cell file junctions in this zone. The only comparisons between zones that yield non-significant results are the inner and radial walls in the cortex. The p-values for all comparisons are given in Table S2.

Zone (i)		Zone (ii)		Zone (iii)		Zone (iv)		Zone (v)	
Distance	Turgor	Distance	Turgor	Distance	Turgor	Distance	Turgor	Distance	Turgor
275	2.95	412	3.46	1456	2.44	2918	4.25	4354	2.96
310	4.02	438	3.46	1675	3.44	3074	4.62	4420	3.32
		520	3.88	1876	3.17	3253	3.4	6246	2.64
		545	3.26	1936	3.64	3260	2.62	7843	2.11
		607	3.98	2057	3.92	3365	2.89	3864	4.12
		678	2.8	2262	3.67		5072	3.95	
		704	2.9	2558	2.82		6702	4.13	
		738	3.1	1579	3.58		3795	3.86	
		751	3.1				4872	3.59	
		777	4.19				7014	4.15	
		808	2.86				4563	4.81	
		812	3.3						
		871	4.03						
		924	2.85						
		953	4.11						
		977	3.28						
		992	1.79						
		1004	3.72						
		1035	3.72						
		1078	2.47						
		1176	3.09						
		1240	3.05						

Table S1: Turgor pressure data as plotted in Fig. 3a. Distances given in μm, turgor in bar. The five developmental zones as defined in De Rybel et al. (2010): (i) meristem, (ii) accelerating elongation zone, (iii) decelerating elongation zone, (iv) mature zone and (v) reference zone.

Tissue	cell-junction	p-value	Significant?
Pericycle	Radial	0.0070	Yes
	Outer	1.15×10^{-5}	Yes
Endodermis	Radial	0.024	Yes
	Inner	1.15×10^{-5}	Yes
	Outer	0.017	Yes
Cortex	Radial	0.096	No
	Inner	0.14	No
	Outer	0.04	Yes
Epidermis	Radial	1.9×10^{-11}	Yes
	Inner	1.44×10^{-5}	Yes

Table S2: Statistical significance of comparison of cell thicknesses Z1A vs. Z1B
